# A Text-Book of Animal Physiology with Introductory Chapters on General Biology and a Full Treatment of Reproduction

**Published:** 1890-01

**Authors:** 


### REVIEW DEPARTMENT.

A Text-Book of Animal Physiology with Introductory Chapters on General Biology and a Full Treatment of Reproduction. For Stduents of Human and Comparative Veterinary Medicine and of General Biology. By Wesley Mills, M.A.M.D.L.R.C.P., (Eng ) Professor of Physiology in McGill University and the Veterinary College, Montreal. New York : D. Appleton & Co., 1889.

To such an extent have recent researches modified the principles of biology that the science of physiology, to which biology is more closely alied than any other science, has undergone a profound change in its methods of investigation and mode of presentment. Human physiology can no longer be viewed as a separate science, the outcome of experiments and observations made upon the human subject, but as the expression of a phase in a long line of scientific conclusions running through the whole range of organized being. For this approach to a distinctly long logical classification of the sciences of hnman comparative physio'ogy, we are, in a special manner, indebted to the rapid progress which the theory of evolution has recently made and its cardiac acceptance by men of scienc as the true means of solving the problem that beset them on every side. Protoplasm is the raw material from which all specifically different organized substances are formed, and the scrutiny of the biologist must be directed to the conditions, both internal and external, which determine primitive homogeneous material to the assumption of the various forms under which we behold it in the complete orders of living beings. Embryology then is the most important branch of physiological enquiry since in elucidating its stages we bring to light truths that have a direct bearing on general physiology, or the animal functions viewed without reference to any single species. This is the true and philosophical view that must be taken of the science of physiology if we would lay its foundation deep and broad enough to support a superstructure that must grow with the daily growth of all kindred branches a natural science. Professor Mills has to be congratulated that he has exhibited in his eminently scientific treatise on physiology, a due appreciation of this trnth, and he has carried out his investigations and established his conclusions in harmony with the leading principles of evolutiou.

Hence, he begins his work with a few considerations on general biology as the student of physiology in this view of the science must be acquainted with thc general properties of living things ere he can properly understand the laws that govern its growths in special directions. The movements of molecules in their homogeneous conditions is the first problem for the student to solve, since a knowledge, however imperfect, of such movements first opens his eyes to the gradual development of cells by cleavage and enucleation. He here obtains the proof of that old scholastic truth *omne vivum ex-ovo*, a truth which after having been accepted for centuries was rejected by the so-called French philosophers of the latter part of the 18th century, and which was for the first time experimentally demonstrated by Professor Tyndale. A knowledge of this and of cognate principles having a reference to the development of organic being irrespective of species, must beget in the miud of the student a serious and reflective mood which will greatiy aid his progress over the difficult and intricate road of inquiry along which the science of physiology undertakes to guide him- He will thus find a delight in proceding by the method which nature has sanctioued, and which leads him from the general to the particular, from the simple to the complex, from the homogeneous to the heterogeneous, through differentations and nilegrations. Coming at last to man he beholds in him the masterpiece of organic life, the crown of that edifice which natnre has built with matchless skill, and yet though the crown, an integral part only of the grand structure which he adorns, human physiology approached in this manner and in this spirit takes on a new interest for the student, and is no longer an isolated line of enquiry. Thus we see that a very decided and most desirable change has taken place in the methods of physiological enquiry, and this we must regard as an explanation for the appearance of so many new hand-books on physiology, and notably for this most welcome one of Professor Mills. Comparative physiology is the only science of animal we can allow, since at no stage of our physiological pursuits can we afford to confine our observation to one species if we desire to gain an exact and radical knowledge of animal functions. It is by such a comparative view of organic life that we can account for the universality of phenomena which are strikingly visible in certain species, but the existenee of which in others is so obscure that we would be disposed to overlook them altogether were we not compelled to infer their existence by the laws of comparison. This proposition receives strength and illustration from the remarks of Professor Mills on the law of periodicity or rhythim in nature. Evcry function of the body is performed in obedience to this law, and were it otherwise the perfect harmony of vital processes would be disturbed. But certain functions, such as muscular contraction, seem to be independent of this rhythmic succession of activity. Yet even muscular contraction, however apparently spasmodic and capricious, is in reality subject to periodicity, for it is opposition to periodicity that begets muscular fatigue and muscular repair takes place during the unconscious hours of sleep, whilst the reign in regularly recurrent movements prevails. We point to this fact merely for the purpose of demonstrating the necessity of the simultaneous study of many forms of animal life in order to fully to understand their import and significance.

Professor Mills has briefly, but very clearly, set forth the current views on organic evolution, and has not hesitated to state that none of them fully accounts for specific differences and variations. Whilst to the immortal Darwin is due the credit of having given us the elements out of which must ultimately come the solution of the difficulty, his attempt to explain the details of variations is a failure. Though Professor Mills, with a modesty that well becomes the truly scientific man, leaves the question still an open one so far as details are concerned, he evidently inclines to look with favor on the Neo-Lamarckranism of Cope and Hyatt as well as on the widely differen: opinions of Romanes, Brooks and Weiseman. Professor Mills' method of dealing with this interesting feature of his work is very clear and logical, and lends atiraction to a deep and difficult question. He is not only a shrewd observer, but an exact thinker, and it is evident from the many ingenious reflections and casual remarks with which his book abounds, that he possesses a logical and well trained mind.

C. M. O'L.

